# Subjective Difficulty Scale in Liver Transplantation: A Prospective Observational Study

**DOI:** 10.3389/ti.2022.10308

**Published:** 2022-03-21

**Authors:** Yuki Kitano, Daniel Pietrasz, Elena Fernandez-Sevilla, Nicolas Golse, Eric Vibert, Antonio Sa Cunha, Daniel Azoulay, Daniel Cherqui, Hideo Baba, René Adam, Marc-Antoine Allard

**Affiliations:** ^1^ AP-HP Hôpital Paul Brousse, Centre Hépato-Biliaire, Université Paris Sud, Inserm U 935, Villejuif, France; ^2^ Department of Gastroenterological Surgery, Graduate School of Medical Sciences, Kumamoto University, Kumamoto, Japan; ^3^ Unité INSERM 1193, Villejuif, France; ^4^ Équipe Chronothérapie, Cancers et Transplantation, Université Paris, Saclay, France

**Keywords:** liver transplantation, difficulty, subjective difficulty, technical difficulty, retransplantation

## Abstract

The predictive value of a subjective difficulty scale (DS) after surgical procedures is unknown. The objective of this study was to evaluate the prognostic value of a DS after liver transplantation (LT) and to identify predictors of difficulty. Surgeons prospectively evaluated the difficulty of 441 consecutive liver transplantations from donation after brain death at the end of the surgery by using a DS from 0 to 10 (“the easiest to the hardest you can imagine”). DS was associated with severe morbidity. The risk of graft loss at 1 year remained unchanged from 0 to 6 but increased beyond 6. Graft survival and patient survival of group with DS 7–10 was significantly impaired compared to groups with DS: 0–3 or DS: 4–6 but were significantly impaired for the group with DS: 7–10. Independent predictors of difficult LT (DS ≥ 7) were annular segment 1, transjugular intrahepatic portosystemic shunt, retransplantation beyond 30 days, portal vein thrombosis, and ascites. Of them, ascites was a borderline non-significant covariate (*p* = .04). Vascular complications occurred more often after difficult LT (20.5% vs. 5.9%), whereas there was no difference in the other types of complications. DS can be used to tailor monitoring and anticipate early complications. External validation is needed.

## Graphical Abstract

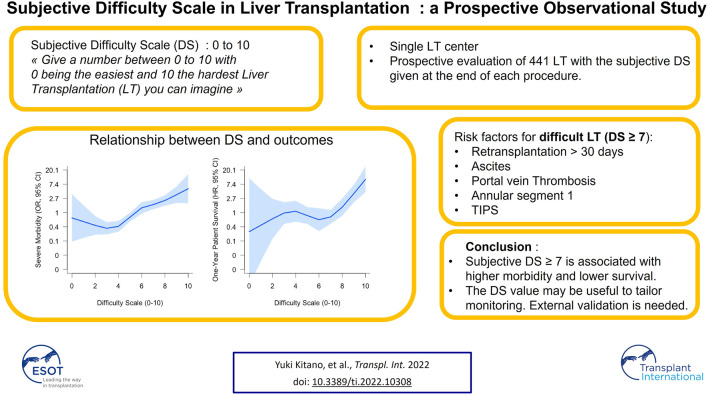

## Introduction

The difficulty in achieving a surgical procedure dramatically varies from one patient to another, independently of its intrinsic complexity (1–5). Several difficulty scoring systems have been published in various surgical fields. These scores are usually built using surrogates of difficulty like blood loss or operation time (3, 6–8), or after selecting risk factors according to expert opinions (4, 5, 9).

This study focused on the technical difficulty of liver transplantation (LT) and proposed a different approach for assessing difficulty. Surgeons prospectively evaluated the difficulty by using a scale ranging from 0 to 10, according to their feeling at the end of the LT.

The prognostic value of such a subjective difficulty scale (DS) is unknown. Balance of Risk (BAR) and Survival Outcomes following Liver Transplantation (SOFT) scores are two validated tools that predict early survival after LT (10, 11). Both include donor and recipient pretransplant variables and cold ischemia time as the unique intraoperative parameter. We hypothesized that the performance of these scores could be improved by adding a subjective DS.

The objectives of this study were to test the impact of DS on outcomes and its added value with regard to validated prognostic models. Lastly, we aimed at identifying preoperative variables that predict difficult LT.

## Patients and Methods

### Study Population and Design

This study included all consecutive patients who underwent LT with a full liver graft from donation after brain death from January 2015 to March 2019 at the Paul Brousse Hospital, Villejuif, France. Every LT involved a fellow, defined here as a “junior” surgeon, and an attending defined as a “senior” surgeon. At the end of each LT, junior surgeons were in charge of entering intraoperative data into a dedicated online questionnaire, including a DS item. Junior surgeons were to give a number ranging from 0 to 10 (0 being the “easiest LT that you can imagine” and 10 being the “most difficult LT you can imagine”).

From October 2018 until the end of the study period, both senior and junior surgeons were asked to evaluate the DS, blinded for the evaluation of each other.

LTs without DS were not included. Donor variables were retrieved from the Cristal database of the Agence de la Biomédecine, the French national agency in charge of organ allocation. The design of this study was discussed and approved at our weekly institutional research meeting. This study was achieved in accordance with French legal requirements and the Declaration of Helsinki. Before surgery, patients provided their written consent according to which they permit that data obtained during standard health care can be used for scientific purposes.

### CT Scan Review

Pretransplant CT scans were reviewed by YK, blinded for outcomes and DS value. The presence of the following items was assessed:

- annular segment I, defined as a complete inferior vena cava encirclement by hypertrophic caudal lobe.
- significant spontaneous portosystemic shunt (SPSS) ≥ 7 mm in diameter.

### Technical Aspect of Liver Transplantation

Total hepatectomy was achieved with caval preservation and transient porto-caval anastomosis in most recipients. The caval anastomosis was done according to the three vein-piggy back technique (12). In the case of huge native liver, or annular segment 1, caval replacement was the preferred option. Portal inflow was obtained with a porto-portal termino-terminal anastomosis. PV thrombectomy was performed when necessary. In the case of a large spleno-renal shunt, left renal ligation or reno-portal anastomosis were decided according to the possibility of using the native portal vein (13). Extra-anatomical PV anastomosis was considered as the last option. For arterial reconstruction, hepatic artery with gastro-duodenal bifurcation was the option of choice.

### Postoperative Management

Initial immunosuppression comprised a triple-drug regimen of tacrolimus, mycophenolate mofetil, and corticosteroid. Steroid boluses were used to treat moderate to severe acute rejection episodes after histological documentation. In selected cases, everolimus was introduced to enable early withdrawal of tacrolimus (14). An injected CT scan on day seven was performed routinely to detect vascular abnormalities (15). The post-transplant management and monitoring were done according to our local protocols regardless of the DS.

### Statistical Analysis

All statistical analyses were performed using R version 3.5.1.

#### General overview

Our analysis followed 6 steps:

Step 1: We tested the relationship between DS and severe morbidity and 1-year patient survival.
Step 2: We evaluated the additional predictive value of DS by comparing the performance of BAR and SOFT scores before and after adding the DS.
Step 3: We compared survival according to three levels of difficulty: “easy” (0–3), “intermediate” (4–6), and “difficult” (7–10). Cutoff values to define these categories were arbitrarily chosen.
Step 4: We performed a univariate and multivariate analysis for predicting difficult transplantation.
Step 5: We compared the type of complications according to difficult transplantation.
Step 6: We tested the senior-junior agreement of DS during hepatectomy and implantation.

### Methodology

In step 1, the relationship between DS and severe morbidity and 1-year patient survival was explored by using regression and Cox models, respectively.

DS was treated not as an ordinal variable but as a continuous variable for simplicity. Severe morbidity was defined by at least one grade IIIa event according to the Dindo-Clavien classification (16). Since several individuals have evaluated the DS, we sought for the possibility of subject-specific correlation. We tested whether the variable “individuals evaluating the DS” should be considered as a random or fixed variable (*lremTest* package) in the regression model. No significant random effect for this variable was detected, which led us to abandon mixed effect models. We left the variable “individuals assessing DS” in the logistic regression and Cox models as a covariate for more robustness (*rms* packages). Restricted cubic splines were used to relax from the linearity assumption (17). The assumption of proportionality of the Cox model was verified with Schoenfeld residuals.

In step 2, we evaluated the performance of the models without and with DS by using the Area Under Curve (AUC) and Akaike Information Criterion (AIC).

In step 3, graft survival was calculated from the date of LT. Data were censored at the time of last follow-up. The event of interest for graft survival was death or retransplantation, whereas death was the only event of interest used for patient survival calculation. Of note, for 1-year patient survival calculation, patients who died after 1 year from LT were censored. Survival curves were plotted according to Kaplan-Meier method. Survival probabilities were compared by using the log-rank test (*ggplot2* packages).

In univariate analysis (step 4 and 5), continuous variables were expressed as median (range) and compared with the non-parametric Mann-Whitney test. Categorical variables were evaluated using chi-squared or Fisher exact tests, as appropriate. Variables associated with difficult transplant (*p* < .10) were entered into a multivariate regression model. The final choice of the model was guided according to the lowest AIC.

In step 6, we used the Lin concordance correlation coefficient (18) (*DescTools* package) to assess the agreement between junior and senior surgeons.

## Results

Of the 631 LT performed during the study period, 525 LT met the inclusion criteria, i.e., a whole liver graft from donation after brain death. After excluding LT without available DS (*n* = 84, 16%), we obtained a study population of 441 LT, including a primary LT in 371 cases and retransplantation in the 70 remaining cases. During the study period, 404 patients underwent a single LT, 17 required two LTs, and one patient was transplanted three times, which represents a total of 422 patients.

For our study population, the 3-month and 1-year graft survival were 93% and 87%, respectively. One-year patient survival was 91%. Severe morbidity occurred in 166 (37.6%) LTs. A primary non-function was observed in 16 cases (3.6%).

**FIGURE 1:**
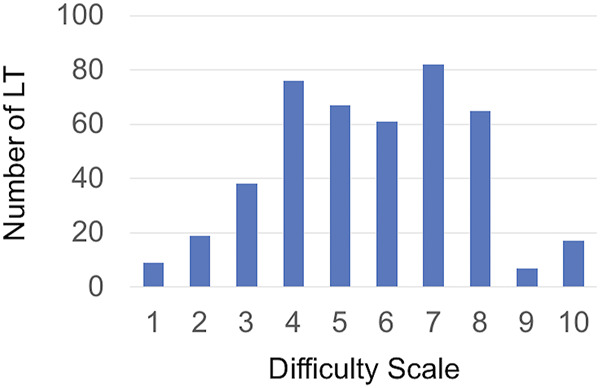
Distribution of DS value across the study population.

The DS was evaluated by twelve junior surgeons. The median value of DS was 6, ranging from 1 to 10. DS was comprised between 0–3, 4-6, and 7–10 in 66 (15%), 204 (46.3%), and 171 (38.8%) LTs, respectively. The distribution of DS values is shown in Figure 1.

### Association Between Difficulty Scale Value and Severe Morbidity and One-Year Survival

**FIGURE 2:**
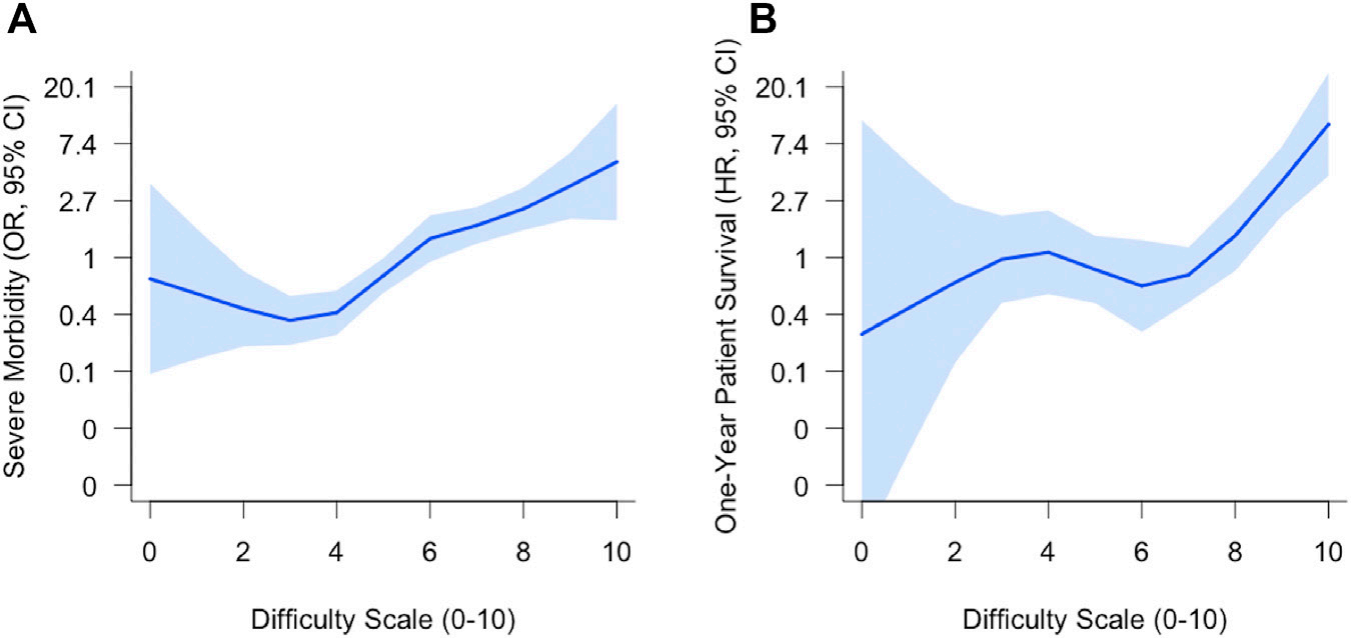
Risk for severe morbidity **(A)** and 1-year patient survival **(B)** according to DS values. Shaded regions indicate 95% confidence bands. HR, Hazard Ratio; OR, Odds Ratio.

As shown in Figure 2, a continuous increase in the risk of severe morbidity as the DS increases was observed. In contrast, the hazard risk of death within the first year remained stable from 0 to 5 and started to increase from 6 to beyond.

### Additional Predictive Value of Difficulty Scale

The predictive value of BAR and SOFT models are given in Table 1. An increase of AUC and a decrease of AIC for all models were observed when adding the DS. The AUC of the models (with and without DS) were compared, and tests were significant for each model, indicating that DS improves the predictive value of each model for severe morbidity, 3-month graft survival, and 1-year graft survival.

**TABLE 1:** Performance of SOFT and BAR models with and without DS for severe morbidity, 3-month graft survival, and 1-year patient survival.

Severe morbidity							
Model	Variables	OR	95% CI	*p*	AUC	AIC	*p* ^a^
One-variable model	SOFT	1.06	1.03–1.09	<.001	.63	545	
Two-variable model	SOFT	1.06	1.03–1.08	<.001	.721	510	
	DS	1.40	1.26–1.57	<.001			<.001
One-variable model	BAR	1.08	1.04–1.12	<.001	.619	549	
Two-variable model	BAR	1.07	1.05–1.13	<.001	.727	510	
	DS	1.48	1.30–1.64	<.001			
**3-months graft survival**							
** Model**	**Variables**	**RR**	**95% CI**	***p***	**AUC**	**AIC**	***p* ^a^**
One-variable model	SOFT	1.02	1.02–1.38	.227	.632	226	
Two-variable model	SOFT + DS	1.02	1.02–1.38	.441	.715	216	<.001
		1.38	1.14–1.70	.001			
One-variable model	BAR	1.03	.96–1.10	.304	.619	227	
Two-variable model	BAR	1.04	.97–1.11	.25	.720	217	<.001
	DS	1.40	1.16–1.72	<.001			
**One-year patient survival**							
** Model**	**Variables**	**HR**	**95% CI**	***p***	**AUC**	**AIC**	***p* ^a^**
One-variable model	SOFT	1.07	1.03–1.11	<.001	.664	407	
Two-variable model	SOFT	1.07	1.03–1.11	.001	.709	397	<.001
	DS	1.34	1.12–1.59	.001			
One-variable model	BAR	1.08	1.2–1.14	.007	.626	412	
Two-variable model	BAR	1.08	1.2–1.14	.008	.701	399	<.001
	DS	1.39	1.17–1.66	<.001			

a Comparisons of AUC, with the roc. test function (pROC, package).

BAR; balance of risk; DS, difficulty scale; SOFT, survival outcomes after liver transplantation; OR, odds ratio.

### Survival According to DS 0–3 vs. 4–6 vs. 7–10

**FIGURE 3:**
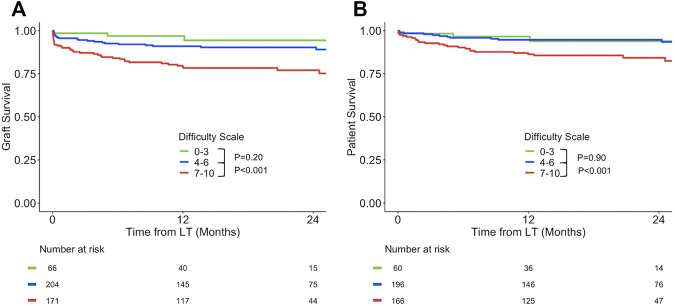
Kaplan-Meier curves for graft survival **(A)** and patient survival **(B)** according to DS 0–3 vs. 4–6 vs. 7–10.

Graft survival and patient survival are reported in Figure 3. Graft survival of the group with DS ≥ 7 was significantly lower than graft survival with DS: 4-6 or DS: 0–3. Graft survival rates were 79% (95% CI: 73–85%), 91% (95% CI: 87%–95%), and 96% (95% CI: 93%–100%) at 1 year for the group DS: 7–10, DS: 4–6, and DS: 0–3, respectively. There was no difference between the two other groups DS 0–3 and DS: 4–6. Similar findings were observed for patient survival. One-year patient survival rates were 85% (95% CI: 82%–92%) in group DS 7–10 vs. 95% (95% CI: 92%–98%) and 97% (95% CI: 92%–100%) in the group with DS: 4–6 and DS: 0–3, respectively.

### Predictive Factors of Difficult LT (DS ≥ 7)

Univariate analysis is shown in Table 2. Transplant recipients with DS ≥ 7 had ascites, annular segment 1, PV thrombosis, or portal cavernoma more often. A previous transjugular intrahepatic portosystemic shunt (TIPS) was more present in this group. This group was also more likely to include ReLT > 30 days. The final multivariate model included five independent predictors of transplant with DS ≥ 7: previous TIPS (OR: 2.67 [1.06–7.11]), ascites (OR1.64 [1.07–2.51]), Portal Vein thrombosis (OR 2.17 [1.20–3.95]), annular segment 1 (OR 6.57 [2.71–18.48]), ReLT > 30 days (OR 4.11 [2.18–7.98] Table 3). Of note, ascites was a borderline non-significant variable in this multivariable model.

**TABLE 2:** Risk factors for difficult LT (DS ≥ 7): Univariable and multivariable logistic regression analysis.

Variables	DS < 7	DS ≥ 7	*p*	Multivariate analysis		
	*N* = 270 (range or %)	*N* = 171 (range or %)		OR	95% CI	*p*
Recipient						
Male Sex	184 (68.1)	128 (74.9)	.161			
Age, years	55.0 (15.0–71.0)	53.0 (12.0–71.0)	.300			
BMI, kg/m^2^	25.2 (15.4–45.7)	25.1 (11.4–46.1)	.741			
MELD score at transplant	19.0 (6.0–40.0)	19.0 (6.0–40.0)	.516			
ICU at the time of transplant	53 (19.6)	29 (17.0)	.564			
Pretransplant dialysis	12 (4.44)	10 (5.85)	.663			
ReLT beyond 30 days	17 (6.30)	32 (18.8)	<.001	4.11	2.18–7.99	<.001
TIPS in place	8 (2.96)	16 (9.41)	.007	2.68	1.06–7.12	.02
Combined Kidney transplant	16 (5.93)	12 (7.02)	.797			
Explant weight, g	1,295 (400–6,290)	1,315 (435–3,665)	.532			
Pretransplant TACE	53 (19.6)	31 (18.2)	.812			
Night time (10 pm–6 am)	43 (15.9)	28 (16.4)	>.99			
Donor						
Male sex	142 (52.6)	100 (58.5)	.266			
Age, years	60.0 (6.00–91.0)	57.0 (14.0–93.0)	.318			
BMI, kg/m^2^	24.7 (13.8–51.3)	24.2 (14.6–41.0)	.595			
Weight of the graft, g	1,332 (700–2,425)	1,400 (685–2,795)	.168			
GW/recipient BW ratio	1.8 (.7–4.3)	1.8 (.8–5.9)	.601			
Explant weight/recipient BW ratio	1.7 (.7–10.5)	1.7 (.6–6.9)	.965			
Pretransplant CT scan						
Ascites^a^	103 (39.0)	95 (56.2)	.001	1.64	1.07–2.51	.04
Annular segment 1	6 (2.27)	25 (14.9)	<.001	6.58	2.71–18.49	<.001
Annular segment 1 and Piggy Back caval anastomosis	3 (1.1)	17 (10.1)	<.001			
Portosystemic shunt	120 (45.5)	116 (69)	<.001			
Portal vein thrombosis	25 (9.5)	38 (22.6)	<.001	2.17	1.20–3.95	.01
PVT Yerdel 1–2^b^	25 (9.5)	30 (17.5)	<.001			
PVT Yerdel 3	0 (0)	8 (4.8)				
Scoring systems						
BAR	8 (1–22)	8 (1–22)	.571			
D-MELD	1,050 (162–5,312)	1,064 (153–3,400)	.387			
SOFT	9 (3–36)	12 (0–45)	.004			
ET-DRI	1.47 (.95–2.86)	1.44 (.97–2.71)	.938			

BAR; balance of risk; BMI, body mass index; BW, body weight; D-MELD, Donor age X MELD, score; ET-DRI, European Transplant—Donor Risk Index; GW, graft weight; ICU, intensve care unit; MELD, Model for end-stage liver Disease; PVT, portal vein thrombosis; RBC, red blood cell; SOFT, survival outcomes following liver transplantation; TACE, transarterial chemoembolization; TIPS, transjugular intrahepatic portosystemic shunt.

a Ascites was defined regardless of its volume, according to intraoperative finding at laparotomy.

b Yerdel classification (30).

() indicates range for continuous variables and % for categorical variables.

**TABLE 3:** Oberved probabilities for difficult LT (DS ≥ 7) according to the number of risk factors (Annular segment 1, ReLT after 30 days, Ascites, Portal vein thrombosis, TIPS).

Observed probability of DS ≥ 7	
No. Factor	No. DS ≥ 7/overall number
0	46/177 (26%)
1	59/169 (35%)
2	45/65 (69%)
3+	18/21 (86%)

Observed proportions of difficult transplant according to the number of factors are given in Table 3. It ranges from 26% to 86% in transplant without risk factors and at least three risk factors.

### Complications Associated With Difficult Liver Transplantation

The type of surgical complications, according to LT difficulty DS < 7 vs. DS ≥ 7, is shown in Table 4. A higher proportion of vascular complications was observed after difficult LT (20.5% vs. 5.9%; *p* < .001). In contrast, there was no difference in the other types of surgical complications between the two groups. However, the proportion of renal failure tends to be higher in the difficult LT group (borderlin significance).

**TABLE 4:** Complications according to DS.

Type of complications	DS < 7	DS ≥ 7	*p*
	*N* = 270	*N* = 171	
Early allograft dysfunction^a^	57 (21.1%)	49 (28.7%)	.091
Vascular complications^b^	16 (5.9%)	35 (20.5%)	<.001
Biliary complications^c^	9 (3.3%)	5 (2.9%)	>.99
Hemorrhage^d^	31 (11.5%)	24 (14.0%)	.520
Infection	71 (26.3%)	56 (32.7%)	.177
Renal failure^e^	18 (6.7%)	21 (12.3%)	.064

a According to Olthoff et al.

b Thrombosis or stenosis of the hepatic artery, the portal vein or caval anastomosis diagnosed on imaging regardless of the management.

c Stenosis or biliary fistula.

d Hemorrhage requiring laparotomy or hematoma on imaging requiring transfusion.

e Stage III acute kidney injury (KDIGO Classification).

### Agreement Between Junior and Senior Surgeons

**FIGURE 4:**
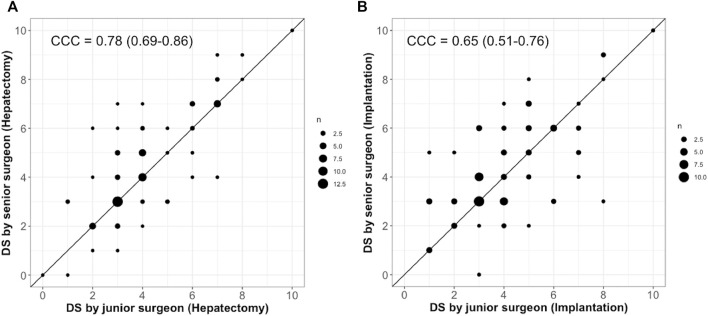
Agreement of DS between junior and senior surgeons for hepatectomy **(A)** and implantation **(B)**.

The DS values given by the junior and senior are given in Figure 4. Diameters of points vary according to the number of evaluations. Points distributed on the diagonal line corresponds to perfect agreement. Points above the diagonal lines indicate that LT was considered more difficult by the senior surgeon, whereas points below refer to harder transplant from the junior point of view. Overall, the agreement was satisfactory. The concordance coefficient correlations (95% CI) were .65 (.51–.76) and .78 (.69–.86) for hepatectomy and implantation, respectively.

## Discussion

The technical difficulty is inherently subjective. In previous studies, the technical difficulty in surgery was assessed by using various surrogates. The originality of our study was to prospectively evaluate the difficulty according to the surgeon’s subjective feeling at the end of the transplantation.

We observed that DS correlates with morbidity and even 1-year survival. The importance of intraoperative factors to improve the predictive ability of pretransplant models has been recognized (19, 20). Adding the sole DS was sufficient to improve two validated pretransplant models, namely the BAR and SOFT scores, which means that DS should be not be used in lieu of these models but in conjunction.

As expected, the DS was associated with some objective variables like duration of surgery or transfusion volume, already known to impact outcomes (21, 22). The main strength of the DS is to reflect some subjective predictors of outcomes such as the surgical field exposure, the quality of tissues, and the easiness to achieve vascular or biliary anastomosis, which cannot be captured by usual metric tools. The DS can be seen as a summary of the numerous factors of difficulty, all contributing directly or indirectly to outcomes. This latter point may explain the predictive value of the DS.

The risk of death within the first year started to sharply increase beyond 6, suggesting that this cutoff value of seven carries a relevant clinical meaning. Five independent factors of “difficult” transplant were identified. Of them, late retransplantation is not a surprising finding. Adhesions, sometimes filled by portal hypertension, and modified anatomical landmark makes ReLT more challenging than primary transplantation (23, 24). A complete encirclement of the retrohepatic inferior vena cava is known to increase the difficulty and the risk of total hepatectomy with caval preservation (25). Preexisting TIPS is also associated with an increased risk of bleeding during total hepatectomy, especially in cases of misplacement (26). PV thrombosis may compromise the portal inflow, essential for graft function recovery. In most cases, eversion thrombectomy is sufficient to restore a sufficient portal flow. In the presence of a complete thrombosis of the PV and superior mesenteric vein, other more technically demanding strategies are needed to obtain adequate portal perfusion. The impossibility of restoring sufficient portal flow may force to consider technically demanding strategies, which consist of anastomosing the graft PV to the recipient superior mesenteric vein, gastric, choledochal varices, or left renal vein (27, 28).

Identifying “difficult” transplants with pretransplant variables yields some logistics advantages. Recipient laparotomy should begin as early as possible to limit cold ischemia time. DS highlights some technical difficulties such as annular segment 1 or portal vein thrombosis and may serve to better define the surgical strategy before LT. Complex transplantation may also require a team of two experienced surgeons. It may also guide the graft choice and avoid the combination of a marginal graft and complex transplantation associated with poor results (29).

The DS may also be of interest in the early post-transplant period. Some patients after “technically easy” LT are likely good candidates for enhanced recovery protocol, whereas recipients with high DS may potentially benefit from tailored monitoring, including daily Doppler and systematic CT scan. However, the possibility to tailor monitoring according to DS remains a hypothesis, and a more refined difficulty scale (evaluating each step, for example) might be a more efficient approach to anticipate outcomes.

The DS proposed here is prone to biases. An important variation in the evaluation according to experience, surgical skills, and timing of surgery could be expected. A surgeon’s “feeling at the end of LT” can be affected by innumerable variables, including the type of procedure, time of day, surgeon or assistant exhaustion or mood, issues with anesthesia, instruments, staff personnel, and many other factors, some even unrelated to surgical or medical aspects. As a result, the same case, potentially with the same outcome, could be subjectively evaluated by the surgeon differently in contrasting circumstances. In addition, the agreement across centers may not be warranted, depending on recruitment, number of cases, and type of disease treated. We also observed acceptable agreement between the senior and junior surgeon evaluations, suggesting that DS keeps a reasonable degree of reproducibility, despite its subjectivity. Discordant values in the DS were mainly observed in the intermediate range of difficulty, whereas “difficult” and “easy” were less subject to disagreement. The present study carries some limitations, in addition to its monocentric nature. The DS has not been evaluated in 16% of LT. We decided not to use multiple imputations because DS is the primary variable of interest. The comparisons of the study population with the group of LTs without DS showed significant differences for junior surgeons but neither for recipient characteristics nor intraoperative data.

The DS did not evaluate specifically for total hepatectomy and graft implantation in the whole cohort. A pretransplant DS would also have been helpful to test predictive variables and study the discrepancy between pre- and post-transplant DS. Validation of the DS prognostic value and the risk factors for complex transplant on an independent cohort is necessary to test the reproducibility and the relevancy of the DS in routine.

In conclusion, end-transplant DS predicts morbidity and 1-year survival after liver transplantation. Its value may be helpful to adapt monitoring and facilitate the early diagnosis of complications.

## Data Availability

The raw data supporting the conclusion of this article will be made available by the authors, under reasonable request.

## Abbreviations

BAR, balance-of-risk; DS, difficulty scale; ET-DRI, Eurotransplant Donor Risk Index; ICU, intensive care unit; LT, liver transplantation; MELD, Model for End-stage Liver Disease; PV, Portal vein; ReLT, Retransplantation; RBC, red blood cells; TACE, transarterial chemoembolization; TIPS, transjugular intrahepatic portosystemic shunt; SOFT, survival outcomes following liver transplantation.
